# Identification of High Platelet Reactivity Despite ADP P2Y _12_ Inhibitor Treatment: Two Populations in the Vasodilator-Stimulated Phosphoprotein Assay and Variable PFA-P2Y Shapes of Curve

**DOI:** 10.1055/a-2075-7979

**Published:** 2023-06-07

**Authors:** Cyril Mariethoz, Emmanuelle Scala, Elena Matthey-Guirao, Jean-Benoît Rossel, Francisco Javier Gomez, Francesco Grandoni, Carlo Marcucci, Lorenzo Alberio

**Affiliations:** 1 Faculty of Biology and Medicine, UNIL, University of Lausanne, Lausanne, Switzerland; 2 Dept. of Anaesthesiology, Lausanne University Hospital (CHUV), Lausanne, Switzerland; 3 Division of Haematology and Central Haematology Laboratory, Lausanne University Hospital (CHUV), Lausanne, Switzerland; 4 Center for Primary Care and Public Health (Unisanté), University of Lausanne, Lausanne, Switzerland

**Keywords:** ADP P2Y _12_ receptor, clopidogrel, response, laboratory data, VASP

## Abstract

**Introduction** Response to ADP P2Y _12_ receptor inhibition by clopidogrel can be evaluated by various techniques. Here, we compared a functional rapid point-of-care technique (PFA-P2Y) with the degree of biochemical inhibition assessed by the VASP/P2Y _12_ assay.

**Methods** Platelet response to clopidogrel was investigated in 173 patients undergoing elective intracerebral stenting (derivation cohort *n* = 117; validation cohort *n* = 56). High platelet reactivity (HPR) was defined as PFA-P2Y occlusion time <106 seconds or VASP/P2Y _12_ platelet reactivity index (PRI) >50%.

**Results** In the derivation cohort, receiver operator characteristics analysis for the ability of PFA-P2Y to detect biochemical HPR showed high specificity (98.4%) but poor sensitivity (20.0%) and a very low area under the curve (0.59). The VASP/P2Y _12_ assay revealed two coexisting platelet populations with different levels of vasodilator-stimulated phosphoprotein (VASP) phosphorylation: a fraction of highly phosphorylated, inhibited platelets and another of poorly phosphorylated, reactive platelets. Analysis of the PFA-P2Y curve shape revealed different types, categorized by time of occlusion (<106 seconds, 106 to 300 seconds, >300 seconds), and pattern (regular, irregular, and atypical). Noteworthy, curves with late occlusion and permeable curves with an irregular or atypical pattern correlated with VASP-PRI >50% and smaller sizes of the inhibited platelet subpopulation. Considering the PFA-P2Y shape of the curve for the detection of HPR improved sensitivity (72.7%) and preserved specificity (91.9%), with a rather high AUC (0.823). The validation cohort confirmed the VASP/P2Y _12_ assay data and the usefulness of considering the PFA-P2Y curve shape.

**Conclusion** In patients treated with acetylsalicylic acid and clopidogrel for 7-10 days, the VASP/P2Y _12_ assay reveals two coexisting subpopulations of differentially inhibited platelets, whose relative sizes predict global PRI and distinct PFA-P2Y curve patterns, indicating incomplete clopidogrel efficacy. The detailed analysis of both VASP/P2Y _12_ and PFA-P2Y is necessary for optimal detection of HPR.

## Graphical abstract

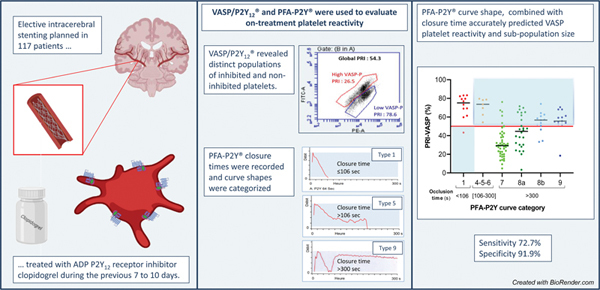

## Introduction

High platelet reactivity (HPR) or low platelet reactivity (LPR) under clopidogrel treatment could lead to an increased rate of ischemic 1 2 or bleeding events, 3 4 respectively. Several assays to evaluate treatment response are used in clinical and research settings, such as light transmission aggregometry (LTA) induced by ADP (LTA-ADP), point of care (POC) devices such as PFA-P2Y, Multiplate, VerifyNow, and the flow cytometry-based VASP/P2Y _12_ assay. While LTA-ADP is considered the functional gold standard to evaluate the response to ADP P2Y _12_ receptor inhibition, 5 6 the VASP/P2Y _12_ test analyzing the VASP phosphorylation state is the biochemical one. 7

Resistance to clopidogrel (HPR), empirically defined as <10% reduction in platelet LTA-ADP, is expected to be observed in about 10 to 15% of patients. 8 Several mechanisms mediating clopidogrel resistance have been described. 9 Clopidogrel is metabolized into its actives compounds by the hepatic cytochrome 450 2C19 (CYP2C19), whose activity is modulated by genetic polymorphisms 10 and numerous pharmacological interactions. 11 Besides metabolism, a decreased gastrointestinal adsorption of clopidogrel might affect the plasmatic concentration of its active metabolites. Finally, intrinsic mechanisms mediating clopidogrel resistance may be platelet P2Y _12_ receptor variability, increased release of ADP, and/or upregulation of other platelet activation pathways. 12 Although HPR under clopidogrel treatment has been linked to an increased risk of thrombotic events and a recent network metanalysis concluded that guided anti-ADP P2Y _12_ treatment is safer and effective than unmonitored, 13 routine testing for clopidogrel resistance is currently not advocated in cardiovascular medicine, 14 15 with the possible exception of patients undergoing elective carotid artery stenting. 16

At our center, interventional radiologists routinely perform platelet function testing 7 to 10 days after having started double antiplatelet treatment and in case of resistance to clopidogrel wait for an additional week, increase clopidogrel dosage, or switch to ticagrelor before proceeding to stent insertion.

Flow cytometry measurement of the phosphorylation state of vasodilator-stimulated phosphoprotein (VASP) specifically detects biochemical inhibition of the ADP P2Y _12_ receptor and correlates well with the degree of functional platelet inhibition, 5 thus permitting the identification of low responders to clopidogrel therapy. 17 Its use for the monitoring of clopidogrel therapy has been associated with an improved outcome. 18 VASP/P2Y _12_ results correlate well with LTA-ADP ( *r* = 0.66 to 0.90 depending of the concentration of ADP used for LTA), and therefore, this assay is considered as the biochemical gold standard. 7

INNOVANCE Platelet Function Analyzer employing the PFA-P2Y cartridge is a functional rapid POC test using whole blood. This system exhibits only a moderate correlation with LTA-ADP ( *r* = −0.51) 19 and VASP/P2Y _12_ ( *r* = −0.41), 19 explaining the significantly different prevalence of HPR and LPR depending of the assay used. 4

Our aim was to compare the diagnostic performance of VASP/P2Y12 and PFA-P2Y and investigate reasons for discordant results.

## Methods

### Patient Population

Between January 2018 and April 2021, we investigated platelet reactivity in 117 consecutive patients treated with acetylsalicylic acid (ASA) 100 mg and clopidogrel 75 mg o.d. (without loading dose) during 7 to 10 days for whom elective intracerebral stenting, for severe cerebral arterial stenosis, was planned. The observations made in this original derivation cohort were verified in a validation cohort of 56 patients investigated between May 2021 and January 2023.

### Assessment of Platelet Reactivity

Blood was drawn into 3.8 mL collection tubes (Sarstedt, Nümbrecht, Germany) with buffered citrate (3.8%, pH 5.5). In order to evaluate platelet response to P2Y _12_ -inhibitors, we performed following commercial assays according to the manufacturer's instructions: INNOVANCE PFA-P2Y test cartridge on a PFA System (Siemens Healthcare Diagnostics Products GmbH, Marburg, Germany) and VASP/P2Y _12_ (BioCytex, Marseille, France). The latter assay was performed on an ACCURI C6 flow-cytometer (Becton Dickinson, BD, Franklin Lakes, NJ, United States). In the VASP/P2Y _12_ assay, the platelet reactivity index (PRI) was calculated from the mean fluorescence intensity of VASP as published. 20 An in-house validation showed an excellent reproducibility of the results performed on the same patients in two distinct laboratories (slope 1.0, 95% CI: 0.88–1.20; intercept: 11%, 95% CI: 22–3%; *p* = 0.86; *n* = 26). HPR despite treatment with P2Y12-inhibitors was defined as: PFA P2Y occlusion time <106 seconds 21 or VASP-PRI >50%. 4

### Analysis of Vasodilator-Stimulated Phosphoprotein Phosphorylation State

We observed that the distribution of individual platelets on the VASP dot plots is highly heterogeneous, highlighting platelets with various degrees of phosphorylation. Particularly in the presence of intermediate PRI values, two distinct populations of highly and poorly phosphorylated platelets can be distinguished (see Results: Double Platelet Populations in the VASP/P2Y _12_ Assay). In this study, the relative sizes and PRI of these subpopulations were calculated separately by manual gating, performed by a laboratory technician, confirmed by the head technician, and verified by an experienced hematologist in case of doubt.

### Analysis of PFA-P2Y Results

We classified PFA-P2Y results according to closure time and shape of the blood flow curves (see Results: Various PFA-P2Y curve types, and Supplementary Material Figures S1 and S2 ). The timing of the occlusion provides three different categories: early occlusion (<106 seconds), late occlusion (between 106 and 300 seconds), and permeable (no occlusion within 300 seconds). The shape of the curve also provides three categories: regular curves (linear evolution), irregular curves (variability in the evolution of the curve characterized by a “saw-tooth” appearance), and atypical curves (either regular or irregular curves, further characterized by an initial strong flow reduction and a sudden marked secondary increase of the intradevice blood flow). Based on these criteria, we defined 10 categories. Early occlusive curves were subdivided into type 1 (regular), type 2 (irregular), and type 3 (atypical). Late occlusive curves were subdivided into type 4 (regular), type 5 (irregular), and type 6 (atypical). Permeable curves were subdivided into type 7 (regular), type 8 (irregular), and type 9 (atypical). Type 8 curves were further subdivided into category 8a if the curve pattern tended toward a permeable profile (i.e., the overall blood flow remained above 50% of the initial maximal flow) and category 8b if the curve tended toward an occlusive profile (i.e. the blood flow dropped below 50% of the initial maximal flow). Of note, type 9 curves showed a near occlusion of the intradevice blood flow, followed by a sudden increase of flood flow and a persistence of permeability.

### Statistics

Distribution of data was established by the Kolmogorov–Smirnov test. Continuous variables are described as median, interquartile range (IQR), and minimal–maximal range. Categorical variables are described as numbers and percentages. Pearson's coefficient (r) was calculated for correlation of continuous variables. Cohen's kappa (κ) was used to assess agreement between PFA-P2Y and VASP/P2Y12 in classifying platelet response to clopidogrel (Interpretation of κ: <0 no agreement; 0–0.2 slight; 0.21–0.40 fair; 0.41–0.60 moderate; 0.61–0.80 substantial; 0.81–1.0 almost perfect). 12 To assess the ability of PFA- P2Y to predict HPR defined by a VASP-PRI >50%, receiver operator characteristics (ROC) were analyzed with comparison of the Youden J Index and the area under the curve (AUC). Group means were compared using two way ANOVA with post-hoc Tukey test. *p* < 0.05 was considered statistically significant. Data visualization and statistical analyses were performed using GraphPad Prism (GraphPad Software Inc., California, United States), MedCalc Statistical Software, version 15.11.0 (MedCalc Software bvba, Ostend, Belgium), and Stata (StataCorp LLC, Texas, United States) as appropriate.

## Results

### Derivation Cohort (January 2018–April 2021)

We investigated 117 patients treated with ASA 100 mg and clopidogrel 75 mg o.d. for 7 to 10 days prior to elective intracerebral stenting. Seventy-five (64%) patients were female. The median age was 55.7 years (IQR: 47.4–63.6; range: 19–92). Laboratory values, comorbidities, and comedications are summarized in Table 1 .

**Table 1:** Patient characteristics of the derivation ( *n* = 117) and validation ( *n* = 56) cohorts

	Unit	Derivation cohort	Validation cohort
Personal characteristics		*n* = 117	*n* = 56
Age	Year (median; IQR)	55.7 (IQR: 47.4–63.6)	62.4 (IQR: 53.7–71.0)
Sex	Women (%)	64	50
Laboratory values			
Hematocrit	L/L (median; IQR)	40 (IQR: 37–42)	41 (IQR: 39–43)
Leucocytes	10 ^9^ /L (median; IQR)	6.8 (IQR: 5.7–8.6)	7.1 (IQR: 5.7–8.2)
Platelets	10 ^9^ /L (median; IQR)	249 (IQR: 207–304)	256 (IQR: 229–306)
Creatinine		71 (IQR: 62–81)	69 (IQR: 60–88)
Comorbidities			
Diabetes	Number of patients	14	2
Hypertension	Number of patients	31	18
Hypercholesterolemia	Number of patients	22	16
Hypothyroidism	Number of patients	6	1
Comedications			
Antidiabetics	Number of patients	14	2
Antihypertensive	Number of patients	24	14
Angiotensin converting enzyme (ACE) inhibitors	Number of patients	7	4
Statins	Number of patients	22	16
Beta-blockers	Number of patients	10	8

Abbreviation: IQR, interquartile range.

#### Mismatches Between PFA-P2Y and VASP/P2Y _12_ Results

**Fig. 1.**
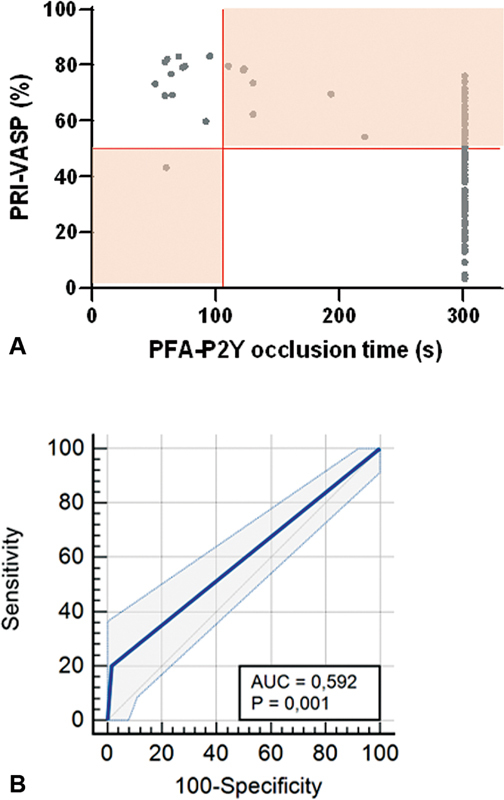
Mismatches between PFA-P2Y and VASP/P2Y _12_ assays. **Panel A:** Distribution of PRI values obtained in the VASP assay with the corresponding occlusion times in the PFA-P2Y system. **Panel B:** ROC curve analysis of the ability of PFA-P2Y occlusion time of 106 seconds to identify HPR as defined by VASP-PRI >50%. For visualization purposes, the cut-off values for HPR in the VASP assay (PRI >50%) and PFA-P2Y test (<106 seconds) have been marked with red lines, separating the graph in four areas. The areas representing a mismatch between VASP-PRI and PFA-P2Y occlusion time for the identification of HPR are highlighted in orange. HPR, high platelet reactivity; PFA, platelet function analyzer; PRI, platelet reactivity index; ROC, receiver operating characteristic; VASP, vasodilator-stimulated phosphoprotein.

The median PFA-P2Y closure time was >300 seconds, with a range from 51 to >300 seconds. According to the definition of HPR on the PFA-P2Y (occlusion time <106 seconds), 12 patients (10%) were identified as HPR ( Fig. 1 , Panel A, left half). Seven (6%) had a closure time between 106 and 300 seconds and 98 patients (84%) presented a permeable PFA-P2Y curve. As for the VASP/P2Y _12_ assay, the median PRI for the 117 patients was 47% (IQR: 29–63; range: 3–88). According to the definition of HPR in this assay (VASP-PRI > 50%), 55 patients (47%) were identified as having clopidogrel resistance ( Fig. 1 , Panel A, upper half).

Of note, in only 72 patients out of 117 (61.5%), there was an agreement between the two assays. Eleven patients (9.5%) were classified as showing HPR and 61 (52%) were classified within therapeutic window (WTW) by both assays. Noteworthy, 45 out of 117 patients (38.5%) presented a mismatch between PFA-P2Y and VASP/P2Y _12_ results. Forty-four patients out of the 55 (80%) classified as HPR by VASP assay were not identified as such by the PFA-P2Y occlusion time ( Fig. 1 , Panel A, orange upper right area). One patient out of the 12 (8%) classified as HPR with PFA-P2Y occlusion time was not identified as such using VASP flow cytometry ( Fig. 1 , Panel A, orange lower left area). Cohen's coefficient of agreement κ was only 0.19 ± 0.06 (95%CI: 0.075–0.3).

Accordingly, ROC analysis for the ability of the PFA-P2Y occlusion time <106 seconds to detect HPR defined by a VASP-PRI >50% showed a very low Youden index (0.18) and AUC (0.59) with a sensitivity of 20.0% and a specificity of 98.4% ( Fig. 1 , Panel B).

#### Double Platelet Populations in the VASP/P2Y _12_ Assay

**Fig. 2:**
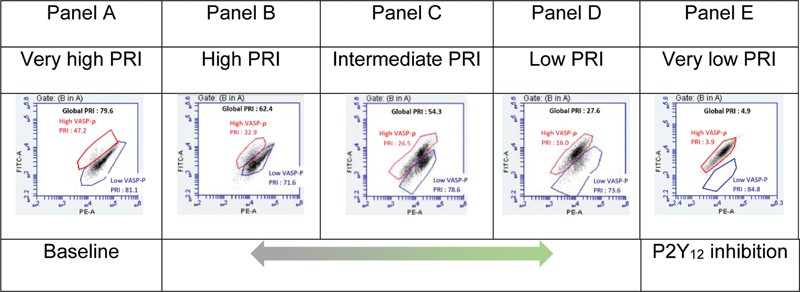
Double platelet population detected by the VASP/P2Y _12_ assay. Platelets inhibited by clopidogrel (defined as “high VASP-P population”) are highlighted in red. Uninhibited platelets (either at baseline or resistant to clopidogrel) are named “low VASP-P population” ( *blue* ). Global PRI, based on all the platelets, is in black. The *x* -axis of the dot-plots represents the platelets (identified by a PE-labelled anti-CD61 antibody) and the *y* -axis describes the level of phosphorylation of the VASP protein. CD, cluster of differentiation; PE, R-Phycoerythrin; FITC, fluorescein isothiocyanate; PRI, platelet reactivity index; VASP, vasodilator-stimulated phosphoprotein.

Close inspection of the raw flow cytometric dot plots revealed the presence of two coexistent platelet populations ( Fig. 2 ), distinct in their VASP phosphorylation levels. Highly phosphorylated platelets (named “high VASP-P population”) represent inhibited platelets (i.e., they show a low PRI). Weakly phosphorylated platelets (low VASP-P population) represent highly reactive platelets (i.e., they show a high PRI).

At baseline, when platelets are not treated with ADP P2Y _12_ receptor inhibitors (or in case of complete resistance to clopidogrel), the PRI of the sample is very high and the large majority of the platelets are concentrated in the low VASP-P population ( Fig. 2 , Panel A). Reciprocally, when platelets are fully responsive to ADP P2Y _12_ receptor inhibitors, the global PRI is very low and almost every platelet is in the high VASP-P population ( Fig. 2 , Panel E). Noteworthy, we observed various combinations of the two VASP phosphorylation states. In samples with a high PRI ( Fig. 2 , Panel B), the majority of the platelets are in the low VASP-P population, with a consistent minority of inhibited platelets (high VASP-P population). In samples associated with a low global PRI ( Fig. 2 , Panel D), most platelets are gathered in the high VASP-P population, with a smaller low VASP-P, noninhibited population. In samples with an intermediate PRI ( Fig. 2 , Panel C), the sizes of both platelet subpopulations are comparable.

**Fig. 3:**
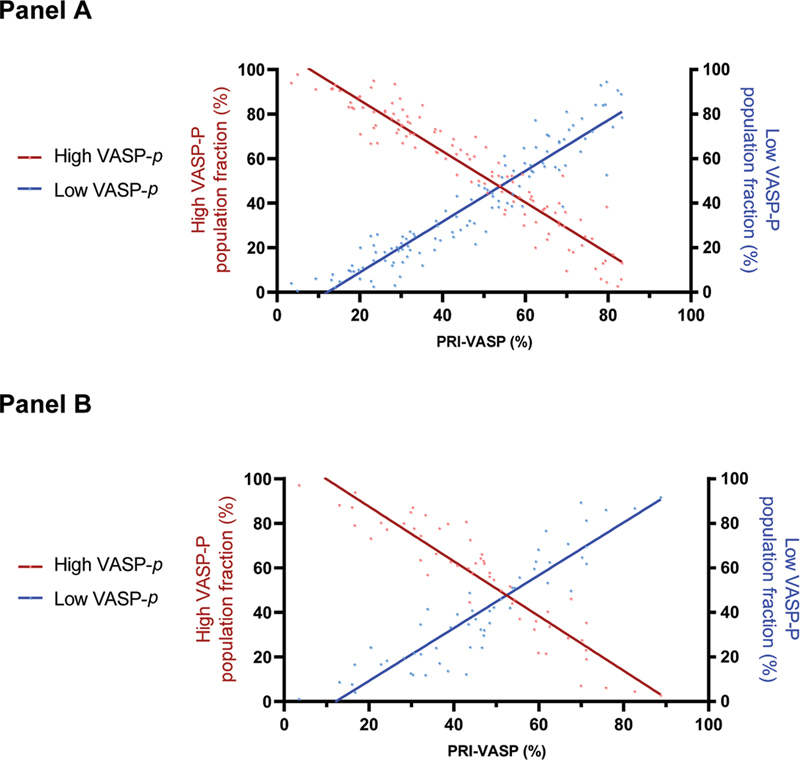
Correlation between global VASP-PRI and the gated fraction of high and low VASP-P platelet populations. **Panel A** : Data from the derivation cohort. **Panel B** : Data from the validation cohort. X-axis: VASP-PRI. Left y-axis: fraction of inhibited platelets (high VASP-P population, see Fig. 2 ), represented by the red dots and curve. Right y-axis: fraction of noninhibited platelets (low VASP-P population, see Fig. 2 ), represented by the blue dots and curve. See text for correlation coefficients. PRI, platelet reactivity index; VASP, vasodilator-stimulated phosphoprotein.

As shown in Panel A of Fig. 3 , the global PRI of the whole platelet population is strongly associated with the reciprocal fractions of inhibited and noninhibited platelets. The correlation between the global PRI of the VASP assay and the fraction of low VASP-P and high VASP-P populations was very strong, with *R* ^2^ equal to 0.90 and 0.89, respectively (both *p* -values < 0.0001).

**Fig. 4:**
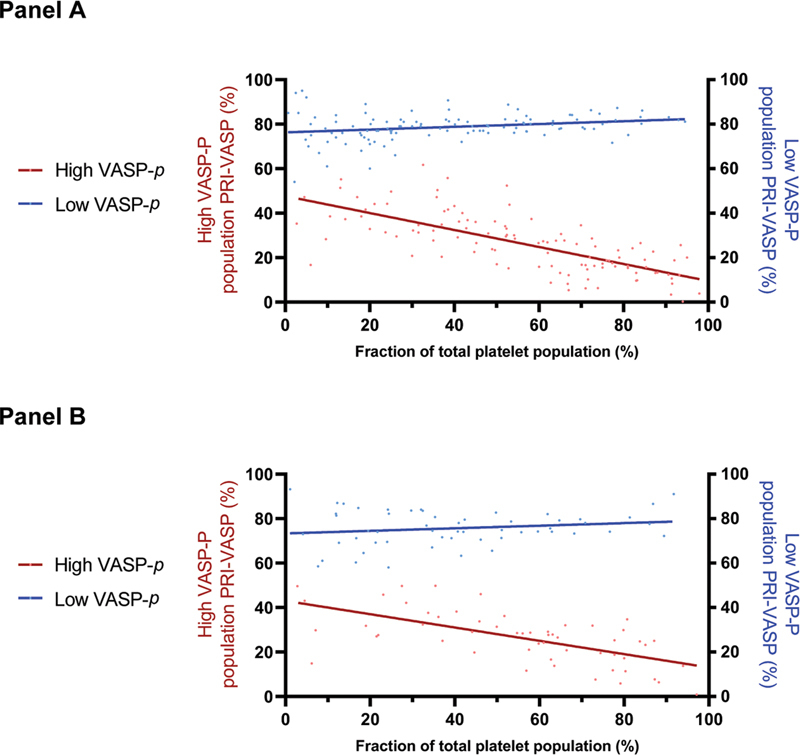
Correlation between PRI of the high VASP-P and low VASP-P sub-populations and their relative percentage. **Panel A** : Data from the derivation cohort. **Panel B** : Data from the validation cohort. X-axis: Platelet population fraction (%). Left y-axis: PRI-VASP of the high VASP-P population (see Fig. 2 ), represented by the red dots and curve. Right y-axis: PRI-VASP of the low VASP-P population (see Fig. 2 ), represented by the blue dots and curve. See text for correlation coefficients. PRI, platelet reactivity index; VASP, vasodilator-stimulated phosphoprotein.

Panel A of Fig. 4 shows that while the PRI of the subpopulation of inhibited platelets correlated with the population size ( *R* ^2^ = 0.52; *p* < 0.0001), this was not the case for the noninhibited platelets ( *R* ^2^ = 0.061; *p* = 0.0084). The PRI of the high VASP-P, inhibited population, showed a median value of 26% (IQR: 17–35; range: <1–62). The PRI of the low VASP-P, noninhibited population showed a median value of 79% (IQR: 76–82; range: 54–95).

#### Various PFA-P2Y Curve Types

**Fig. 5:**
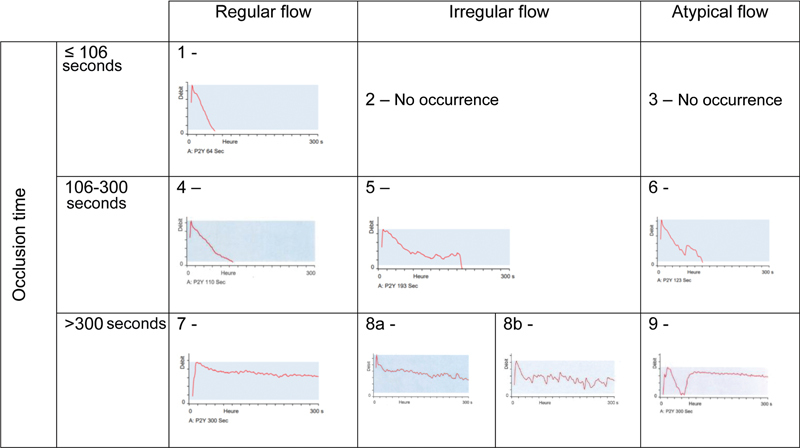
Classification table of the different types of curves on the PFA-P2Y assay. Lines: Classification according to the occlusion time. Columns: classification according to the curve pattern. This classification method shows a partition of the data in ten different PFA-P2Y curve types.

Fig. 5 illustrates the different patterns of PFA-P2Y curves observed in our 117 patients, according to the classification described in the Methods section (Analysis of PFA-P2Y Results). Overall, 10% of the curves were in the group presenting an occlusion of blood flow before the cut-off of 106 seconds (12 curves of type 1 and no occurrence of types 2 and 3). Six percent of the curves presented an occlusion between 106 and 300 seconds (three curves of type 4, one type 5, and three type 6). For analysis purposes, these three categories will be handled as a whole. Finally, in the group of permeable PFA-P2Y tracings (84% of the total), we counted 46 curves of type 7 (39.5%), 26 of type 8a (22.5%), 13 of type 8b (11%), and 13 of type 9 (11%).

#### Association between PFA-P2Y Shape of Curve and VASP-P2Y _12_ Assay

**Fig. 6:**
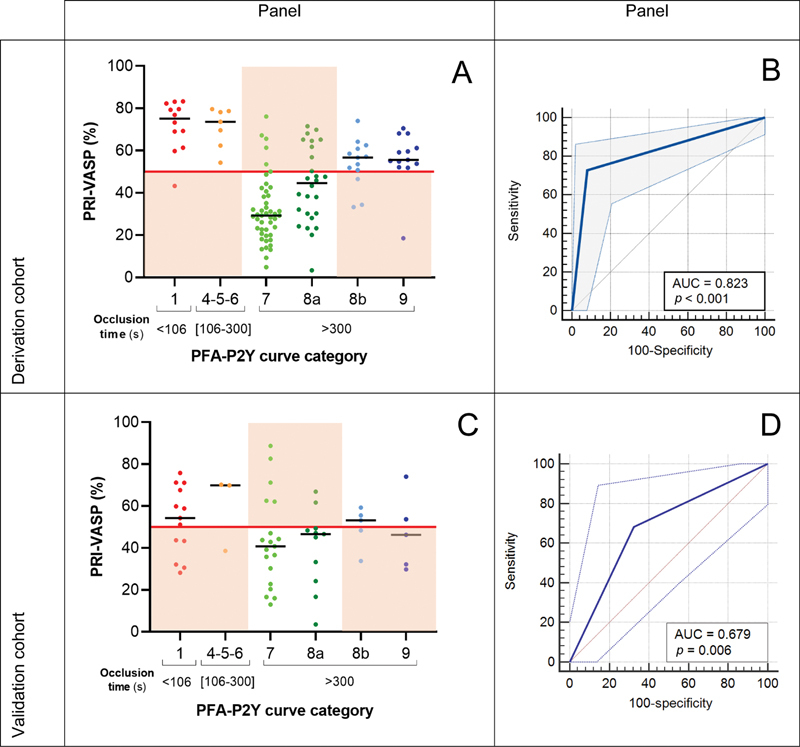
Correlation of PFA-P2Y and VASP/P2Y12 assays considering the PFA-P2Y curve type. Panels A (derivation cohort) and **C** (Validation cohort): PFA-P2Y results classified according to closure time and in-device blood flow pattern. For visibility purposes, the values for each category were represented in a different color, and a red horizontal line was drawn at the VASP-PRI 50% cut-off for the identification of HPR according to VASP flow cytometry assay. The panels related to a mismatch between VASP-PRI and PFA-P2Y occlusion time in the identification of HPR have been highlighted in orange. **Panels B** (Derivation cohort) and **D** (Validation cohort): ROC curve analysis of the ability of PFA-P2Y shape of curve classification to identify high platelet reactivity as defined by VASP-PRI >50%. AUC, area under the curve; HPR, high platelet reactivity; PFA, platelet function analyzer; PRI, platelet reactivity index; ROC, receiver operating characteristic; VASP, vasodilator-stimulated phosphoprotein.

Fig. 6 (Panel A) takes up the elements of Fig. 1 but replaces on the *x* -axis the “PFA-P2Y occlusion time” with the “PFA-P2Y curve category” parameter, thus adding the dimension of the in-device blood flow pattern to the analysis. As summarized in Table 2 , PFA-P2Y curves with an occlusion time of <106 seconds showed a median PRI of 78% (IQR: 69–82; range: 43–83) in the VASP assay, and 11 out of the 12 samples (92%) from this category were classified as HPR. Late occlusive curves (types 4, 5, and 6) showed a median PRI of 74% (IQR: 62–79, range: 54–80). All samples of this category were classified as HPR by VASP assay. The analysis of permeable PFA-P2Y curves was more variegate. Type 7 curves showed a median PRI of 29% (IQR: 21–39, range 5–76) and 40 out of the 46 samples (87%) from this category were classified as WTW by the VASP assay. Type 8a curves showed a median PRI of 45% (IQR: 30–63 range: 3–72) and 17 out of the 26 samples (65%) from this category were classified as WTW. Type 8b curves showed a median PRI of 56% (IQR: 53–65, range: 19–71). Of note, 10 out of the 13 samples (77%) from this category were classified as HPR by the VASP assay. Similarly, type 9 curves showed a median PRI of 56% (IQR: 54–61, range: 19–79) and 12 out of the 13 samples (92%) from this category were classified as HPR by VASP.

**Table 2:** Global PRI and size of high VASP-P population by type of PFA-P2Y curve

PFA curve category		PRI of total sample (%)			High VASP fraction		
Type	*n* (%)	Median	IQR	Range	Median	IQR	Range
1	12 (10.3)	78.1	69.1–82.0	43.3–83.3	0.25	0.12–0.41	0.03–0.65
4, 5, 6	7 (6.0)	73.6	62.4–78.7	54.3–79.6	0.21	0.06–0.39	0.05–0.47
8b, 9	26 (22.2)	56.2	51.9–61.5	18.5–74.0	0.48 a	0.38–0.57	0.21–0.90
7, 8a	72 (61.5)	31.3	23.3–47.4	3.4–76.1	0.78 b	0.63–0.88	0.11–0.99

Abbreviations: IQR, interquartile range; PRI, platelet reactivity index; VASP, vasodilator-stimulated phosphoprotein.

a Significantly different ( *p* < 0.05) from category 1, from category 4, 5, 6 and from category 7, 8a.

b Significantly different ( *p* < 0.05) from category 1, from category 4, 5, 6 and from category 8b, 9.

#### Association between PFA-P2Y Shape of Curve and “High VASP-P Population” Size

**Fig. 7:**
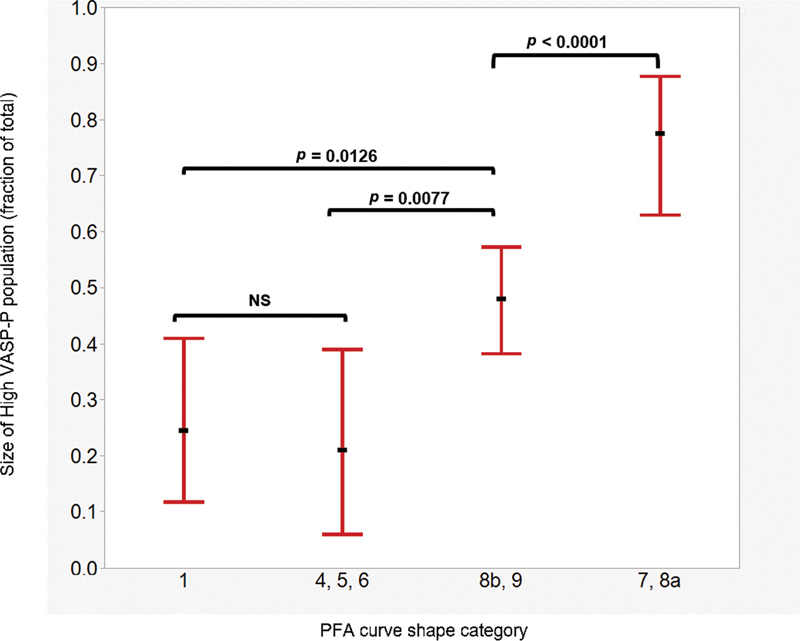
ANOVA with posthoc Tukey test describing the relationship between the fraction of high VASP-P population and PFA-P2Y curve shape category. Category 1: curves with closure time <106 seconds. Categories 4, 5 and 6: curves with closure times between 106 and 300 seconds. Categories 8b and 9: permeable curves with atypical or irregular shapes and flow reduction > 50%. Categories 7 and 8a: permeable curves with regular shapes of irregular with flow reduction < 50%. In categories 1, 4, 5, and 6 only about one quarter of all platelets are inhibited. In categories 8b and 9, around half of the platelet population is inhibited. In categories 7 and 8a, three quarters of platelets are inhibited. Dots represent median, bars represent interquartile range. NS: nonsignificant. *p* < 0.05 is significant.

On the one hand, the different shapes of the PFA curve are associated with the degree of inhibition (as seen in Fig. 6 , Panels A and C), and on the other, the degree of inhibition is associated with the relative size of the high VASP-P population (as seen in Fig. 3 ). Therefore, we compared the size of the high VASP-P, inhibited subpopulation in type 1 curves (the standard definition of HPR by PFA-P2Y) with that of other curve types ( Table 2 and Fig. 7 ). The median fraction of high VASP-P platelets in type 1 curves is only 0.25 (IQR: 0.12–0.41; range: 0.03–0.65). The fraction of inhibited platelets in types 4, 5, and 6 (occlusion after 106 seconds) is similarly low with a median of 0.21 (IQR: 0.06–0.39; range: 0.05–0.47). In samples with curve types 8b and 9 (permeable but irregular and drop of flow below 50% of the maximal value), the fraction of inhibited platelets reaches the half of the entire platelet population (median: 0.48; IQR: 0.38–0.57; range: 0.21–0.90) and is significantly higher than in types 1, 4, 5, and 6 ( *p* = 0.0126). Finally, in curve types 7 and 8a (permeable and flow consistently above 50%), the fraction of high VASP-P platelets was 0.78 (IQR: 0.63–0.88; range: 0.11–0.99), and significantly higher than type 8b and 9 ( *p* < 0.0001).

Considering the above, we propose a new definition of HPR for the PFA-P2Y assay, in which the flow pattern is included as a criterion. All occlusive traces (groups 1, 4, 5, and 6) should be considered as definite HPR regardless of the occlusion time. Permeable traces of types 8b and 9 should be considered as probable HPR and treated as HPR. Only permeable traces of types 7 and 8a indicate adequate platelet inhibition.

According to this new definition of HPR in the PFA-P2Y assay, 45 patients (39%) were identified as HPR (definite or probable). Overall, 40 patients out of the 117 (34%) of the total cohort were classified as HPR by both VASP and PFA-P2Y assays, and 57 (49%) were classified as WTW by both assays. Only 20 out of 117 patients (17%) presented a mismatch in the determination of platelet reactivity category according to PFA-P2Y and biochemical gold-standard VASP. Specifically, 15 patients out of the 55 (27%) classified as HPR by VASP flow cytometry were not identified as such using PFA-P2Y. Five patients out of the 45 (11%) classified as HPR by PFA-P2Y were not identified as such using VASP assay. Cohen's coefficient κ was 0.65 ± 0.069 (95%CI: 0.52–0.79), indicating substantial agreement.

Accordingly, ROC analysis for the ability of the PFA-P2Y occlusion time combined with the interpretation of curve shape to detect HPR as defined by VASP flow cytometry showed high Youden index (0.65) and AUC (0.823), with a sensitivity of 72.7% and a specificity of 91.9% ( Fig. 6 , Panel B).

### Validation Cohort (May 2021–January 2023)

The observations made in the original derivation cohort were verified in a validation cohort of further 56 patients treated with ASA 100 mg and clopidogrel 75 mg o.d. for 7 to 10 days prior to elective intracerebral stenting ( Table 1 ).

#### Double Platelet Population in the VASP/P2Y12 Assay

The coexistence of a double population of inhibited and noninhibited platelets was confirmed ( Fig. 3B and 4B ). As shown in Fig. 3B , the global PRI of the platelet population from the validation cohort is still strongly associated with the reciprocal fractions of inhibited and noninhibited platelets. The correlation between the global PRI of the VASP assay and the fraction of low VASP-P and high VASP-P populations was very strong, with R2 equal to 0.84 and 0.86, respectively (both *p* -values < 0.0001).

#### Association between PFA-P2Y Shape of Curve and VASP-P2Y12 Assay

As shown in Panel C of Fig. 6 , similar to the derivation cohort, PFA-P2Ycurves of the validation cohort patients with an occlusion time <106 seconds showed a median PRI of 54% (IQR: 38–69, range: 28–76) in the VASP-P2Y12 assay and 8 out of the 13 samples (62%) from this category were classified as HPR according to their VASP-PRI. Late occlusive curves (types 4, 5, and 6) showed a median PRI of 70% (IQR: 39–70, range: 39–70). Two out of the three samples of this category (67%) were classified as HPR by VASP/P2Y12 assay. Type 7 curves showed a median PRI of 40% (IQR: 23–62, range: 13–89) and 14 out of the 19 samples (74%) from this category were classified as WTW. Type 8a curves showed a median PRI of 47% (IQR: 24–49, range: 4–67) and 9 out of the 11 samples (82%) from this category were classified as WTW. Type 8b curves showed a median PRI of 53% (IQR: 41–57, range: 34–59). Of note, three out of the five samples (60%) from this category were classified as HPR by the VASP-P2Y12 assay. Similarly, type 9 curves showed a median PRI of 46% (IQR: 31–64, range: 30–74) and two out of the five samples (40%) from this category were classified as HPR by VASP-PRI.

## Explanation of Remaining Mismatches after Consideration of PFA-P2Y Criteria for HPR

### Effect of Hematological Variables on the VASP-P2Y12 Assay

Despite the significant performance improvement of PFA-P2Y when considering the shape of the curve, its ability to predict HPR remains imperfect and there are still some mismatches with VASP flow cytometry ( Fig. 6 , Panels A and C, highlighted in orange). False “responsive” results are the ones presenting a PFA-P2Y curve of types 7 or 8a with a VASP-PRI over 50%, and apparently false “resistant” results are the ones with a PFA-P2Y curve of types 1, 8b, or 9 with a VASP-PRI below 50% according to VASP assay.

Parameters, other than platelet reactivity, that may influence PFA-P2Y results are fibrinogen (reference range: 2–4 g/L), hematocrit (0.40–0.52 L/L), platelet count (150–350 G/L), as well as von Willebrand factor (VWF; 50–150%) and FVIII (65–170%) activities. 22 23 In the derivation cohort, samples with a false “responsive” PFA-P2Y result showed a median hematocrit within the reference range (median: 0.40 L/L; IQR: 0.39–0.43). Platelet count (279 G/L; 246–359) as well as fibrinogen (2.8 g/L; 2.8–3.5) was also within normal limits. There was no measurement of VWF or FVIII for these cases. Samples with a false “resistant” PFA-P2Y result showed hematocrit (0.38; 0.36–0.39) and platelet counts (216 G/L; 205–267) within reference ranges. Fibrinogen (4.6 G/L, 4.5–4.6) was slightly over the norm. Of note, in the single sample with PFA-P2Y closure time <106 seconds despite a low VASP-PRI ( Fig. 6 , Panel A), we observed highly increased values of VWF antigen (285%) and activity (241%), as well as FVIII:C (280%).

Considering both cohorts, we confirmed that PFA-P2Y curves with a “resistant” pattern (types 1 or 4–5–6) despite a VASP-PRI <50% were characterized by high levels of VWF, with a median VWF activity of 221% (IQR: 184–253) and VWF antigen of 171% (IQR: 144–201). On the contrary, PFA-P2Y curves with a “responsive” pattern (types 7 and 8a) despite a VASP-PRI ≥ 50% tended to be associated with higher platelet counts (median 280 G/L) compared to those with a VASP-PRI <50% (median 259 G/L; *p* = 0.0593). Somewhat surprisingly, considering the laboratory variables affecting PFA closure times, we did not find a role for low levels of VWF, hematocrit, or platelets. Finally, for the discordances observed with curves of types 8b and 9, we did not find a particular association with the laboratory data investigated.

### Effect of Comedication on the VASP-P2Y12 Assay

**Fig. 8:**
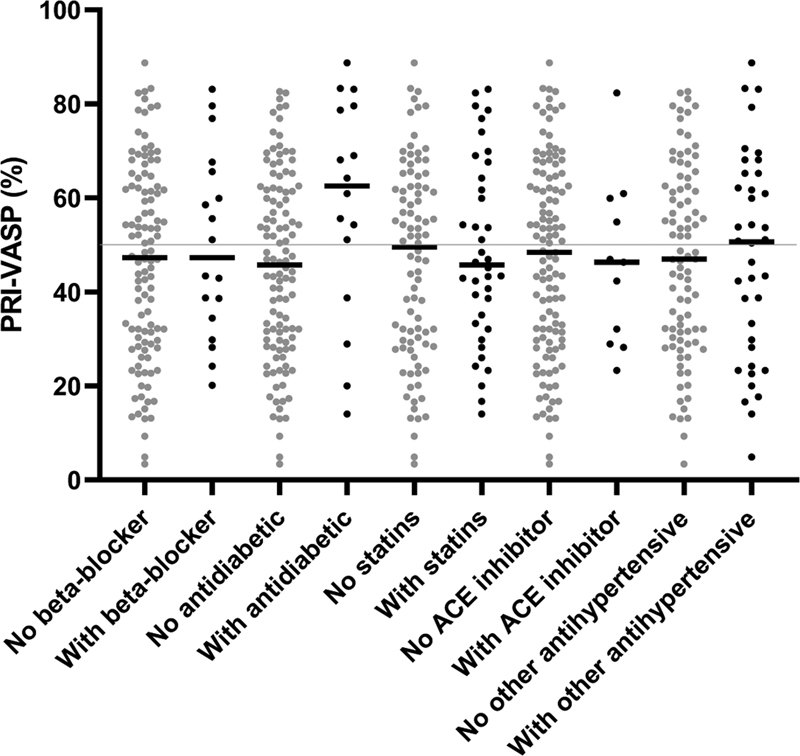
Impact of comedication on VASP/P2Y12 assay's PRI values. Effect of various classes of drugs on VASP-PRI results. Data from combined derivation and confirmation cohorts. *X* -axis: Presence of absence of various drug types. *Y* -axis: VASP-PRI. See text for correlation coefficients. PRI, platelet reactivity index; VASP, vasodilator-stimulated phosphoprotein.

A review of the patient medication at the time of platelet testing was made, including beta blockers, antidiabetic drugs, statins, ACE inhibitors, and other antihypertensive drugs. Medication was documented in 128 (74%) out of the 173 patients of both cohorts ( Fig. 8 ). Eighteen patients (14%) were treated with beta-blockers. Median VASP-PRI of these patients was 47% (IQR: 33–66, range: 20–83) compared to 47% (IQR: 29–62, range: 3–89; *p* = 0.5084) for those without beta-blockers. Sixteen patients (13%) were treated with antidiabetic drugs (metformine, sitagliptin, linagliptin, vildagliptin, semaglutide, empagliflozin, and insulin), showing a median VASP-PRI of 63% (IQR: 42–79, range: 14–89) compared to 46% (IQR: 29–62, range: 3–83; *p* = 0.0140) for those without antidiabetics. Thirty-eight of those patients (30%) were treated with statins; median VASP-PRI was 46% (IQR: 33–65, range: 14–83) and 50% (IQR: 29–62, range: 3–89; *p* = 0.5941), respectively. Eleven patients (9%) were treated with angiotensin converting enzyme inhibitors; median VASP-PRI were 46% (IQR: 29–60, range: 23–82) and 48% (IQR: 30–64, range: 3–89; *p* = 0.8769), respectively. Thirty-eight patients (30%) were treated with other antihypertensive drugs; median VASP-PRI were 60% (IQR: 27–65, range: 5–89) and 47% (IQR: 30–62, range: 3–82; *p* = 0.8009), respectively.

Finally, we found six patients taking drugs known for their potential to interact with clopidogrel ( www.compendium.ch ). Comedications with the potential to reduce the absorption of clopidogrel were amiodarone ( *n* = 2, VASP-PRI 39% and PFA curve shape 7, and 34%/8b); isoniazid ( *n* = 1, VASP-PRI 68% and PFA curve shape 8a); and a triple therapy with lamivudine/abacavir/dolutegravir ( *n* = 1, VASP-PRI 62% and PFA curve shape 8a). The only comedication with the potential to augment the absorption of clopidogrel was cyclosporine ( *n* = 1, VASP-PRI 18% and PFA curve shape 7). One patient was treated with baricitinib (VASP-PRI 30% and PFA curve shape 9).

## Discussion

Our study investigated the platelet response to clopidogrel treatment ongoing for 7 to 10 days in patients for whom elective intracerebral stenting was planned. This allowed examining a homogeneous population in terms of indication, length of clopidogrel treatment, and comedication with Aspirin. Our aim was to compare the diagnostic performance of two tests assessing platelet response to ADP P2Y _12_ receptor inhibition: the functional, simple, and rapid PFA-P2Y and the VASP/P2Y _12_ test assessing the biochemical effect. As depicted in Fig. 1 , we confirmed a poor correlation. 19

### Coexisting Inhibited and Noninhibited Platelets in the VASP/P2Y12 Assay

The double population observed in the VASP assay ( Fig. 2 ) is intriguing and, to our knowledge, has never been reported previously. Over a wide range of global PRI values, a varying fraction of platelets appears to be well inhibited, while the rest is not, which leads us to hypothesize that platelets respond individually and independently of each other to clopidogrel therapy. Of note, the strong correlation between the global PRI and the inversely proportional fractions of reactive and inhibited platelets ( Fig. 3 ) demonstrates that the larger the highly phosphorylated population, the better the antiaggregant effect.

The dichotomous response demonstrated in Fig. 2 may reflect the time dependency of platelet inhibition by clopidogrel and its active metabolites. In fact, in patients in whom clopidogrel treatment was continued in an unaltered posology ( *n* = 7), later testing (after median 7 days, IQR: 6–18, range: 3–28) by the flow cytometric VASP assay revealed a decreased PRI from 74% (IQR: 63–76, range: 43–81) to 43% (IQR: 25–46, range: 12–49; *p* = 0.0015) with an increased population of inhibited platelets (high VASP-P) from 25% (IQR: 19–51, range: 6–62) to 67% (IQR: 61–77, range 46–94; *p* = 0.0022). This is consistent with the observation of a decreasing prevalence of clopidogrel resistance from 31% 24 hours after treatment initiation to 15% at 30 days. 8 The individual differential reactivity of the platelets could be explained by several mechanisms. A high percentage of young, immature platelets at the beginning of clopidogrel treatment has been associated with a higher prevalence of HPR at day 7, independently of CYP2C19 variants. 24 Similarly to the mechanisms described in essential thrombocytemia 25 and diabetes 26 for resistance to Aspirin, which is no longer active after its first hepatic passage, 27 28 a high platelet turn-over may explain a higher fraction of platelets not inhibited by clopidogrel. 29 In fact, since clopidogrel metabolites irreversibly bind to the ADP P2Y12 receptor and are cleared from the plasma within approximately 4 hours, newly generated platelets will not be inhibited till after the next clopidogrel ingestion. On the contrary, we can postulate that a slower transformation of clopidogrel into its active metabolites may result in a shorter window of absent platelet inhibition.

Interestingly, PRI analysis of each platelet subpopulation informs us that noninhibited platelets show a stable PRI, close to 80%, regardless of the size of the population ( Fig. 4 , blue dots). The platelet population responsive to ADP P2Y _12_ receptor inhibitors, however, shows a rising degree of inhibition as its size fraction increases, with a PRI falling from about 40 to 10% between initial and complete platelet inhibition ( Fig. 4 , red dots). This observation might suggest that individual platelets initially respond to clopidogrel in an all-or-nothing manner (the initial PRI drop from around 80 to 40%) and, subsequently, the whole platelet population is progressively further inhibited (the progressive PRI decline from 40% to 10%).

### Variable Flow Pattern of the PFA P2Y Analysis

According to our observations, the presence of a variably responsive, noninhibited platelet population is responsible for the occurrence of *in vitro* platelet aggregation during the PFA P2Y analysis, causing irregular and atypical curve patterns ( Fig. 5 ). This is illustrated by the association of late occlusive curves or permeable curves of types 8b and 9 with high PRI values ( Fig. 6 , Panel A) and lower size of inhibited, “high VASP-P” platelet populations ( Table 2 and Fig. 7 ) compared to permeable curves of types 7 and 8a. We hypothesize that this *in vitro* phenomenon may also occur *in vivo* and cause microthrombotic events.

Existing literature describes a weak correlation between VASP/P2Y _12_ and PFA-P2Y for the detection of HPR. 19 In our study, data demonstrate that PFA-P2Y curves showing a late occlusion (between 106 and 300 seconds) as well as permeable curves with an irregular or atypical pattern (types 8b and 9) are associated with a high PRI, justifying considering the presence of these specific PFA-P2Y curve patterns as supplementary criteria for the detection of HPR. Taking these additional definitions into account, the PFA-P2Y shows a clear improvement in its performance in detecting HPR ( Fig. 6 , Panel B) compared to the use of the 106 seconds cut-off alone ( Fig. 1 , Panel B).

### Discordances between VASP-PRI and PFA P2Y Curve Pattern

We show that abnormal laboratory parameters can affect the PFA-P2Y curve shape leading to mismatches between the two assays. In particular, we observed high levels of VWF in the case of normal PFA-P2Y curves despite VASP-PRI values indicating a good response to clopidogrel (e.g., closure time <106 seconds and VASP-PRI <50%; Fig. 6 , Panels A and C). According to our data, a strongly increased level of VWF is a plausible explanation for this presentation, as VWF is known to sustain *in vitro* formation of clots, even with inhibited platelets. 30 Detecting this type of mismatch may be clinically relevant because this mechanism may occur *in vivo* as well, as we have observed in a patient with clopidogrel treatment failure. 31 On the contrary, thrombocytosis appears to induce permeable curves despite high VASP-PRI values demonstrating HPR. This could be explained by an acquired defect of platelet function or an impaired thrombus formation *in vitro* . 32 33

As for the comedication ( Fig. 8 ), we did not find clues suggesting an impact on VASP-PRI results for statins, β-blockers, ACE inhibitors, and other antihypertensive drugs. Noteworthy, we observed significantly higher VASP-PRI values in patients taking antidiabetics. Since there was no difference in VASP-PRI values among the various antidiabetic drug classes (not shown), we think that this observation may be rather explained by diabetes itself, leading to clopidogrel resistance. 34

### Limitations

The limitations of our study include first the lack of information concerning the outcome of the procedure, in order to assess the predictive value of the presence of an unresponsive platelet population on the clinical course after the elective intracerebral stent insertion. Secondly, the small number of patients within specific patterns of PFA-P2Y curves may constitute a bias in evaluating the ability of these curves to predict HPR.

## Conclusion

We demonstrate that after a 7 to 10 days period of clopidogrel treatment, two populations of differentially inhibited platelets are observed by the VASP/P2Y _12_ assay and that the relative sizes of these two populations predict global PRI. Second, we show that the analysis of the PFA-P2Y shape of curve improves the detection of HPR as defined by the VASP assay. This may be clinically relevant because of the more widespread use of the simple PFA-P2Y POC test, which is performed more rapidly than VASP flow cytometry. Finally, a combined biochemical and functional analysis with VASP/P2Y _12_ and PFA-P2Y is useful for optimal detection of HPR, allowing the identification of cases with a potential thrombotic risk, for example, due to highly increased VWF levels despite an adequate platelet inhibition. A confirmatory study evaluating radiographic and clinical outcomes should be planned.

## Notes

**What is Known About this Topic?**

- The PFA-P2Y point of care test device is widely used to verify platelet inhibition under clopidogrel treatment before elective intracerebral vascular stenting.
- The PFA-P2Y test results show poor agreement with direct measurement of the functional state of the ADP P2Y _12_ receptor using gold-standard VASP/P2Y _12_ assay.

**What Does this Paper add?**

- This work shows that VASP/P2Y _12_ assay detects the co-existence of inhibited and noninhibited platelet populations in patients treated with clopidogrel.
- We found that the relative sizes of these subpopulations predict global platelet reactivity index and flow dynamics in the PFA-P2Y assay.
- Integrating closure time with visual inspection of the PFA-P2Y flow curve improves the detection of high platelet reactivity despite ADP P2Y _12_ inhibitor treatment.
