# Supplementary material for: Identification of High Platelet Reactivity Despite ADP P2Y 12 Inhibitor Treatment: Two Populations in the Vasodilator-Stimulated Phosphoprotein Assay and Variable PFA-P2Y Shapes of Curve
Source: TH Open. 2023 Jun 7;7(2):e143–54. doi: 10.1055/a-2075-7979 (PMC10247305; doi:10.1055/a-2075-7979)
Supplement: Supplementary file 1 — Supplementary Material [file 10-1055-a-2075-7979-s22110046.pdf]

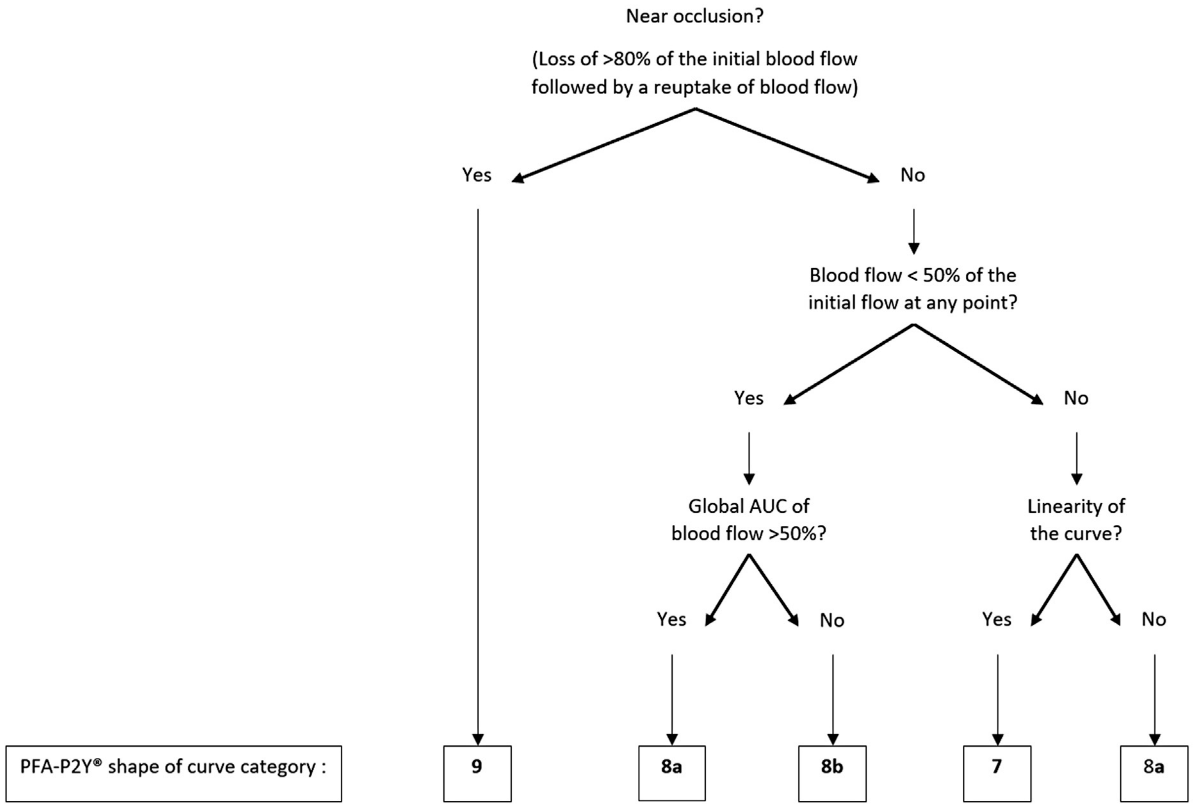

Supplementary Figure S1 Binary decision algorithm for classifying PFA-P2Y curve shape. Lecture from the top to the bottom. Categorization of the PFA-P2Y curve shape by following the appropriate arrow after each question.

 © 2023. The Author(s).

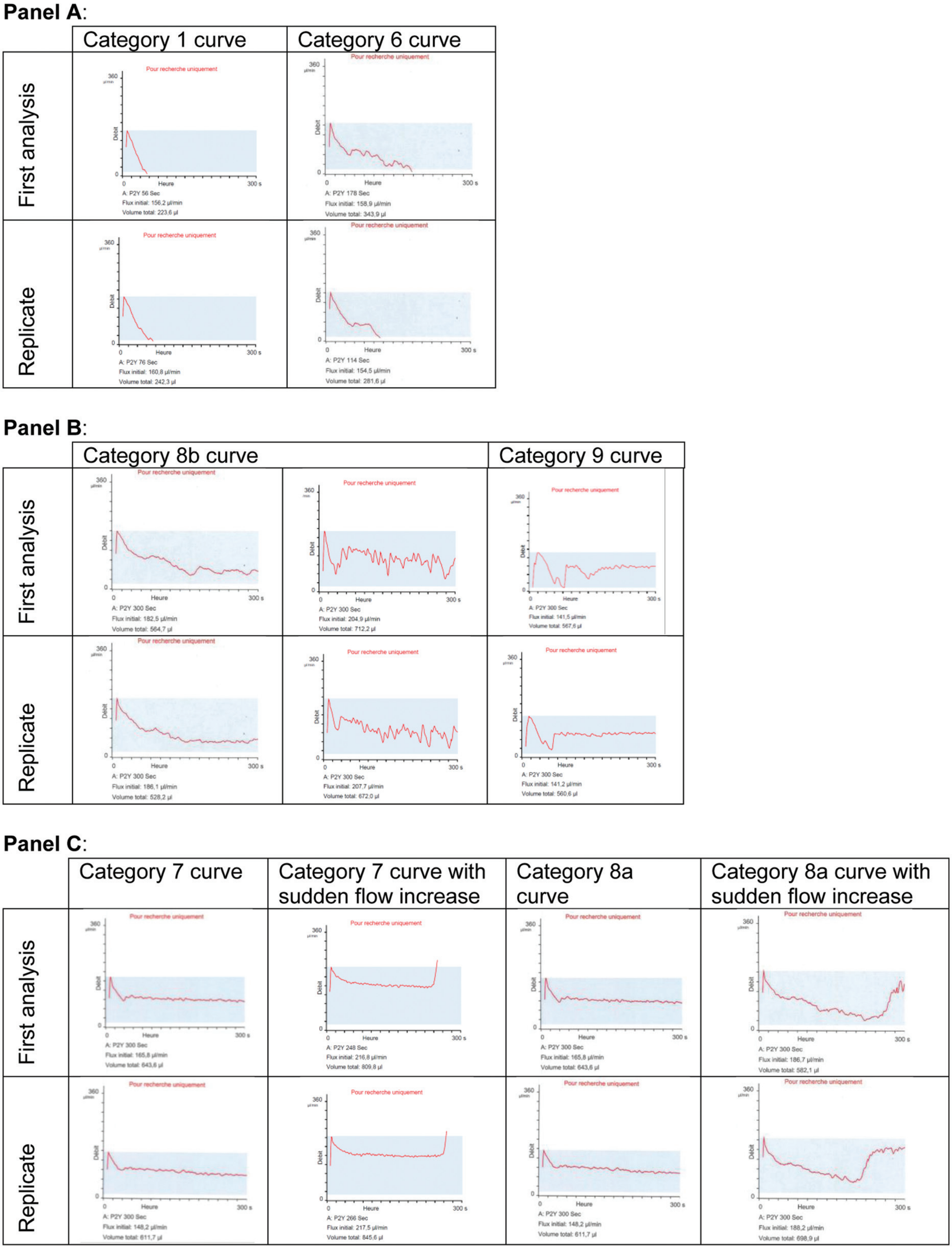

Supplementary Figure S2 Examples of PFA-P2Y curve shape duplicates. **Panel A:** Occlusive PFA-P2Y© curves. **Panel B:** Permeable curves with a high platelet reactivity profile (HPR) profile. **Panel C:** Permeable curves with a profile within therapeutic window (WTW). X-axis: PFA-P2Y curve shape category. *Y*-axis: First PFA-P2Y first analysis and replicate.

 © 2023. The Author(s).
